# The effect of duplex Surface mechanical attrition and nitriding treatment on corrosion resistance of stainless steel 316L

**DOI:** 10.1038/s41598-018-26844-0

**Published:** 2018-05-31

**Authors:** Nana Li, Ning Wang

**Affiliations:** 1 Department of Mechanical Engineering, The Hong Kong Polytechnic University, Hong Kong, People’s Republic of China; 2 College of New Materials and New Energies, Shen Zhen Technology University, Shenzhen, 518055 China

## Abstract

Surface mechanical attrition treatment (SMAT) fabricates a nanostructured layer on the surface of 316L that possesses excellent mechanical properties. In this paper a duplex process combining SMAT with nitriding was performed, then the microstructure, hardness and corrosion properties of the treated materials were investigated by using X-ray diffraction (XRD), transmission electron microscopy (TEM), hardness tests, potentiodynamic polarization measurements, X-ray photoelectron spectroscopy (XPS) and secondary ion mass spectrometry (SIMS). The results showed that this duplex surface treatment can effectively increase the hardness and corrosion resistance of the treated surface both at room temperature and in the simulated steam generator (SG) condition at high temperature and high pressure.

## Introduction

The selection of Ni-based alloys and austenitic stainless steels for core internals of steam generator in nuclear power plants (NPPs)^1^ is driven by the need for good corrosion resistance and mechanical characteristics. Compared with Ni-based alloy, 316L is a cheaper material for SG system, but the low surface hardness and poor corrosion resistance at high temperature have restricted its applications in SG^2–7^. Most material failures occur on the surface and therefore, controlling the surface properties can effectively improve the various materials performance. Grain size refinement of the metallic surface is a promising way to improve the mechanical properties of austenitic stainless steels without changing the chemical composition. SMAT is one of the novel methods^8,9^ which can induce grain refinement into the nanometer scale in the surface layer of bulk samples, which has been successfully applied in a variety of materials, including pure metals, alloys, and intermetallics^10–14^. However, nanocrystalline materials are associated with a high-volume fraction of the grain boundary and a large number of structural defects^15–17^, leading to a significant increase in stored energy which may increase reactivity and accelerate corrosion by forming a large number of micro-electrochemical cells between the grain boundaries and the matrix according to the classic corrosion theory^18,19^. A previous study^20^ also shows that the SMAT process leads to significant residual stress at the surface and degradation of the corrosion resistance of 316L at room temperature.

A practical approach for solving such a difficulty is to introduce the duplex process combining SMAT with nitriding. It is well known that nitriding is an effective process for improving the surface hardness and anti-wear properties of stainless steel. As nanocrystalline steel induced by means of SMAT possesses high chemical activity due to the enhanced atomic diffusion, the duplex SMAT and nitriding treatment lead to the increase in the thickness of nitrided layer and surface hardness for pure Fe and other alloys^8,21–23^.

In this paper, the duplex surface treatments were performed on 316L austenite stainless steel. Then the effects on the microstructure, hardness and corrosion behavior both at room temperature and at high temperature were investigated. The passive film formed on the surface of treated materials in simulated SG chemistries under high temperature and high pressure were also studied.

## Results and Discussion

**Figure 1:**
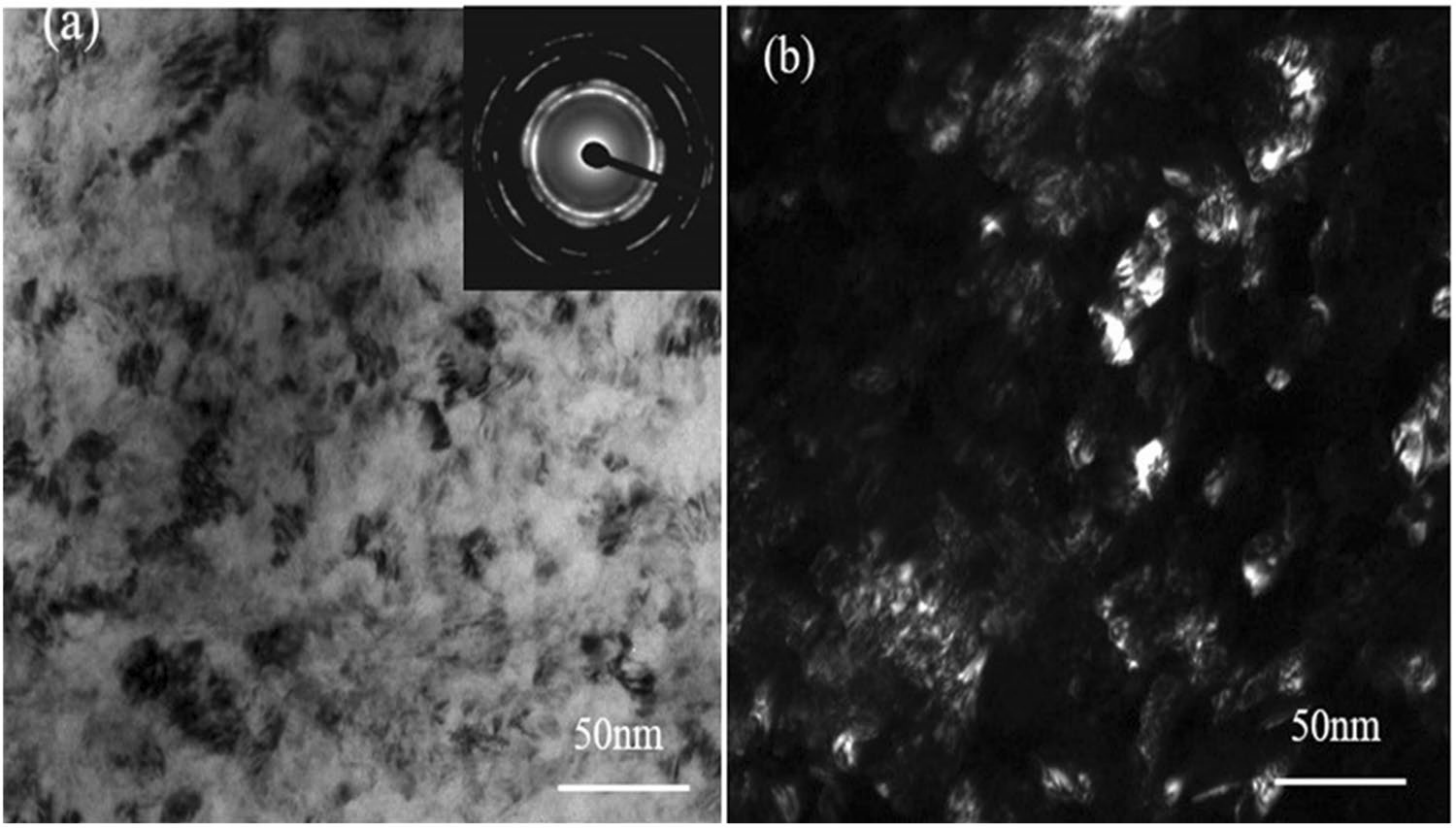
Microstructure of the top surface layer in the sample after SMAT (**a**) A bright-field image with corresponding SAED pattern; (**b**) a dark field image.

The nanocrystalline microstructure of the surface of 316L induced by SMAT was determined by transmission electron microscopy (TEM). Figure 1 shows the TEM images of the top surface layer on the specimen after the SMAT. The corresponding selected area electron diffraction (SAED) pattern exhibits well-defined rings, which illustrate the formation of fine grains with random orientation on the treated surface. This microstructure is characterized by uniformly distributed nanometer-scale grains with mean grain size around 20 nm.

**Figure 2:**
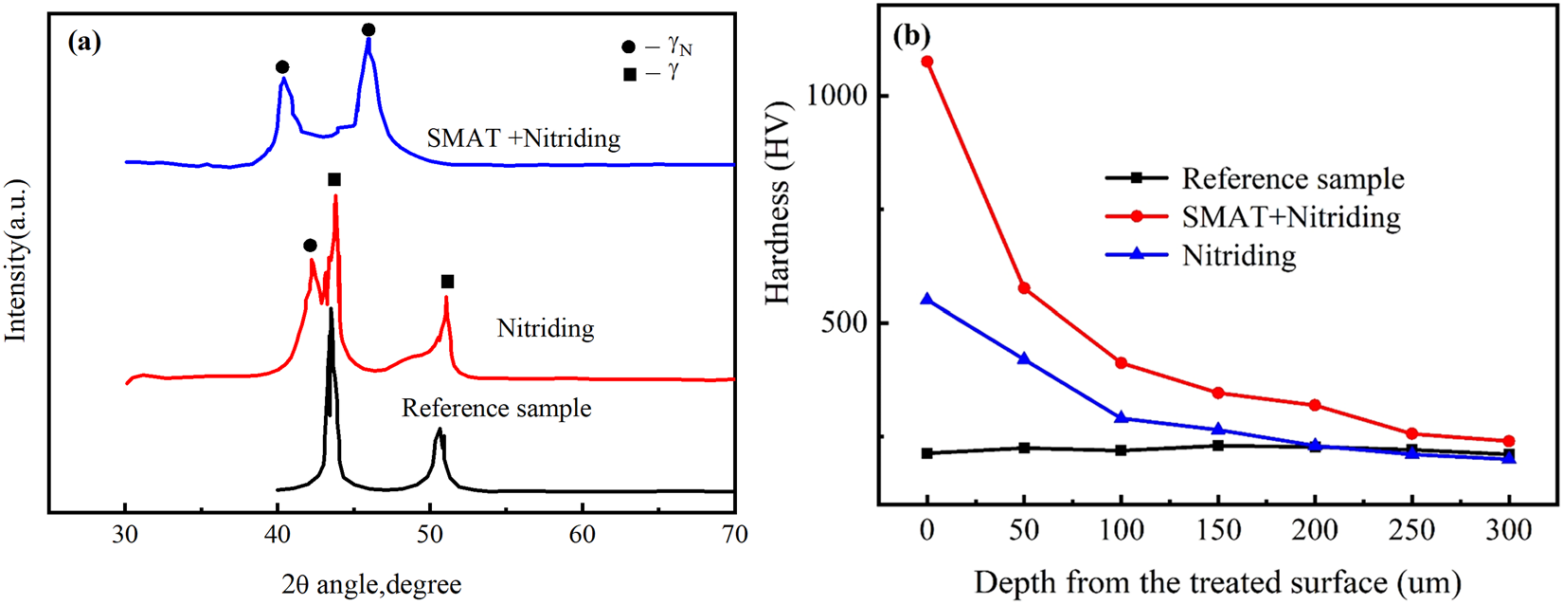
(**a**) X-ray patterns and (**b**) Micro-hardness distribution along depth from the surface for nitrided samples with or without SMAT as the pretreatment.

The XRD patterns of nitrided 316L with and without pre-SMAT treatment at 400 °C are shown in Fig. 2(a). CrN precipitates generally form at high nitriding temperatures (above 450 °C) when Cr start to migrate^24,25^, CrN, which will deteriorate corrosion resistance, was not detected in both samples. The nitrided layer on the sample after duplex treatment was mainly composed of γ-Fe (labeled with γ), as well as small traces of S-Phase (labeled with γ_N_). On the contrary, the XRD pattern of the sample after the duplex SMAT and nitriding treatment at the same temperature was mainly composed of γ_N_. One notable point about the XRD pattern of nitrided samples with pre-SMAT was the negative shift of the γ_N_ peaks to the lower degree. This phase usually forms at low nitriding temperatures, because the solubility limit of nitrogen in austenitic structure was exceeded with increasing of temperature^26^. The explanation for the difference between the γ_N_ peaks positions and intensities for the samples without and with SMAT may be given as the following. Firstly, diffusion of nitrogen atoms was accelerated by the grain boundaries and dislocations formed by SMAT process, which produced plentiful fast diffusion paths for the nitrogen atoms transporting into the substrate. Moreover, these diffused nitrogen atoms concentrated at the surface layer, because the diffusion rate of nitrogen into the matrix was much lower relative to the top nanocrystalline surface layer. Therefore, more γ_N_ formed in the sample with SMAT. The super saturation of nitrogen in the S phase expanded the f.c.c. lattice of the austenite and subsequently the corresponding XRD peaks shifted to lower angles. Figure 2(b) depicts the variation of micro-hardness from the nitride samples without and with SMAT as compared to untreated 316L. It can be seen that the nitriding did change the hardness and hardening layer depth. Furthermore, compared to those of nitrided sample without SMAT, the maximum microhardness was improved dramatically to 1050 HV on the top surface of sample with duplex SMAT and nitriding treatment, which was attributed to a great number of grain boundaries and high defect densities that played a decisive role in the growth of nitrided layer for samples with SMAT.

**Figure 3:**
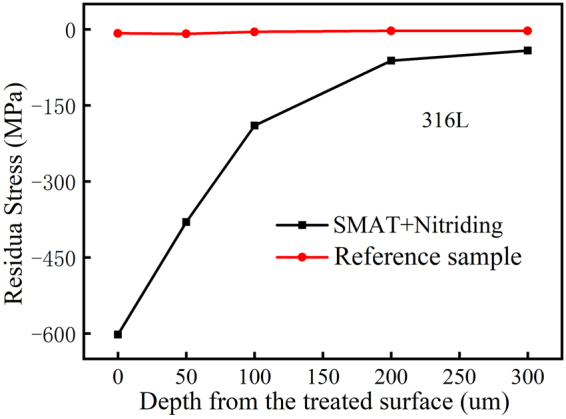
Residual stress depth profiles of 316L before and after SMAT process.

XRD analysis of the nitrided 316L with SMAT showed no martensitic phase or other impurities in the SMAT sample^20^. Residual stress was measured on the specimen before and after SMAT using XRD. It can be seen from Fig. 3 that the residual stress after SMAT for 60 mins reached nearly to 600 MPa compressive stresses.

**Figure 4:**
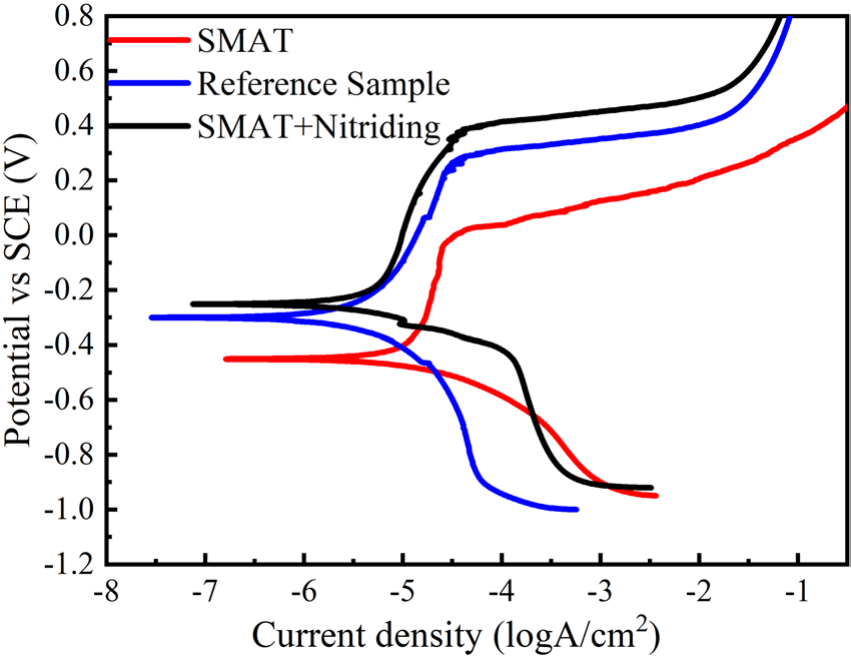
Potentiodynamic polarization curves of 316L at room temperature.

Potentiodynamic polarization curves of reference 316L, nitrided 316L with and without pre-SMAT treatment at room temperature are shown in Fig. 4 and the results were summarized in Table 1.

**Table 1:** Potentiodynmic polarization results at room temperature.

	316L	316L + SMAT	316L + SMAT + Nitriding
I_corr_ (μA/cm^2^)	3.98 ± 0.31	6.56 ± 0.75	2.9 ± 0.23
E_corr_ (*vs* SCE mV)	−300 ± 17	−450 ± 28	−250 ± 15
E_p_ (*vs* SCE mV)	280 ± 20	138 ± 11	350 ± 22

The results suggest that the SMAT process leads to the diminution of corrosion resistance of 316L at room temperature, as the nanocrystalline materials, owing to a high surface energy and defects density, may accelerate corrosion process. However, the corrosion resistance of the 316L samples with the duplex SMAT and nitriding treatment appeared to be improved with respect to the untreated sample and sample treated with SMAT alone at room temperature. The reason comes from two aspects. First, annealing occurs during the nitriding process, resulting in releasing of the strain energy in the surface layer and partly recovering of corrosion resistance. Second, it is well known that the localized corrosion resistance of stainless steels can be effectively improved by adding N to their composition^25–27^. Furthermore, nitrogen can increase local pH during corrosion process by reaction with H^+^ in the solution to form the NH_4_^+^ (N + 4 H^+^ + 3e^−^ –> NH_4_^+^) and leads to facilitate the repassivation.

**Figure 5:**
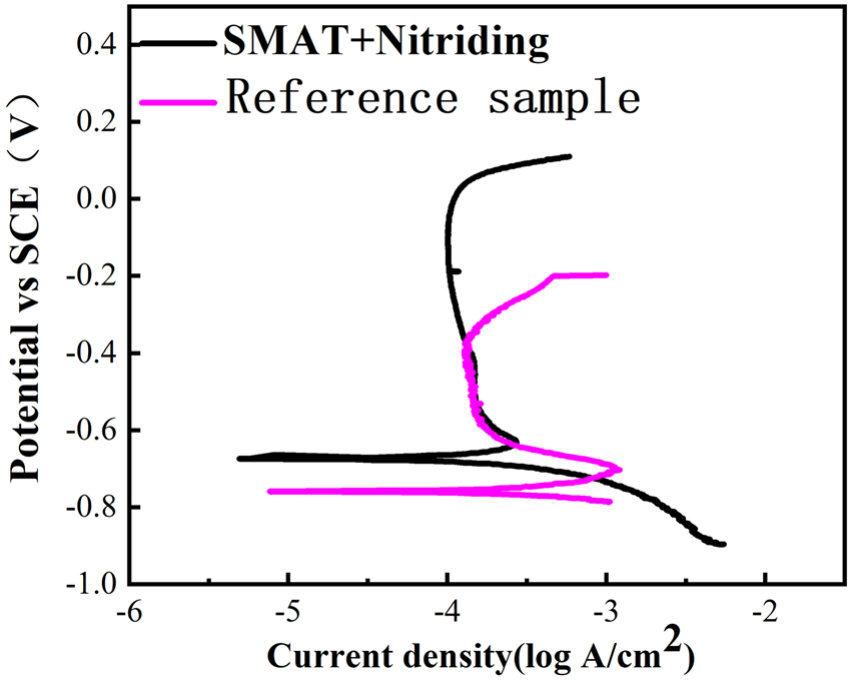
Potentiodynamic polarization curves of 316L in simulated SG condition.

**Figure 6:**
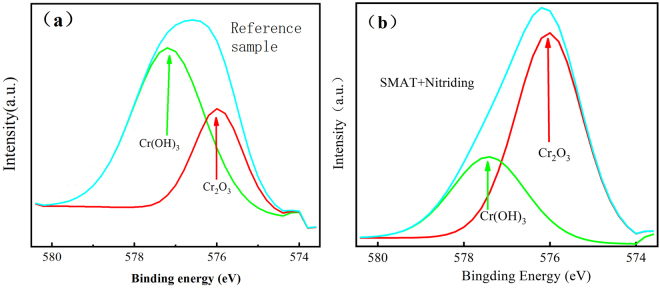
XPS spectra of Cr 2p3/2 at 300 °C simulated SG crevice chemistries on 316L without treatment (**a**) and with treatment (**b**).

**Figure 7:**
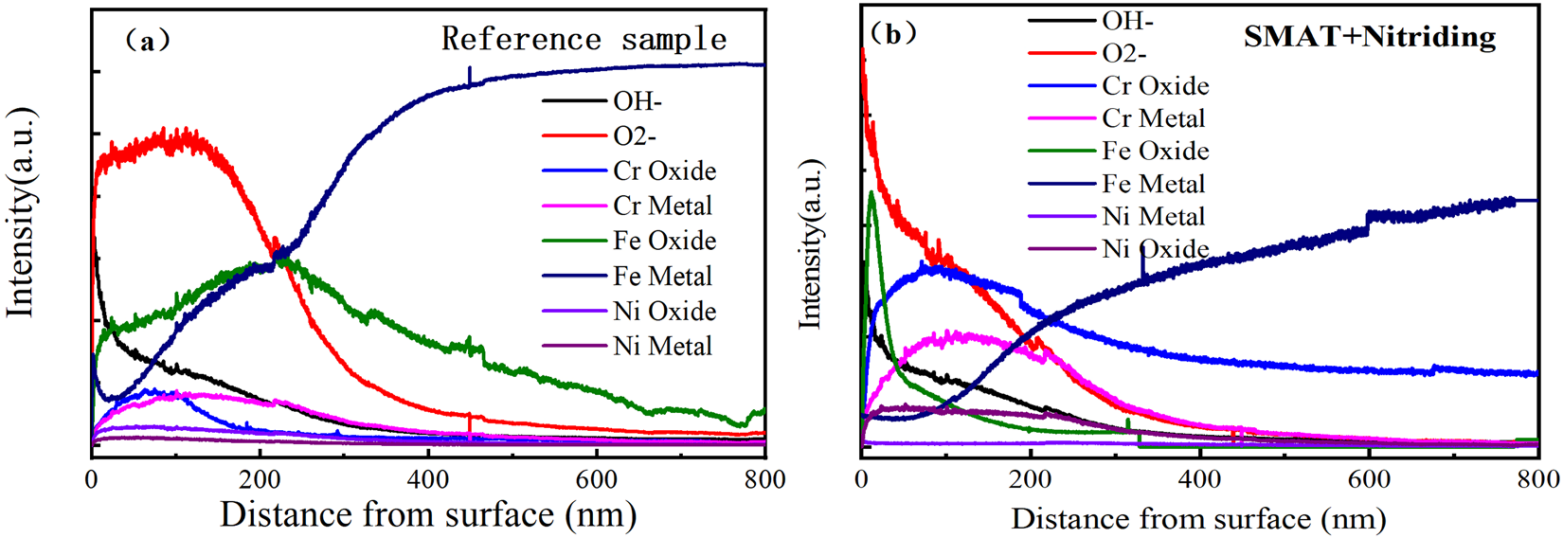
The depth profile of the oxide films formed at 300 °C simulated SG crevice chemistries on 316L without treatment (**a**) and with treatment (**b**).

Potentiodynamic polarization tests were also carried out on 316L in simulated SG crevice chemistries at 300 °C to investigate the corrosion behavior of reference sample and sample with duplex SMAT and nitriding treatment at high temperature. The polarization curve is shown in Fig. 5. It can be seen that both of the samples showed typical characters with small active region followed by a well-defined passive region and transpassive region. The passive region of untreated 316L was approximately from −650 mV (SCE) to −350 mV (SCE), while the passive region of treated sample expanded approximately by 100% from −600 mV (SCE) to 0.0 mV (SCE). The pitting potential shift from −350 mV (SCE) for untreated 316L to 0 mV (SCE) for treated one. Hence, the duplex SMAT and nitriding treatment caused significant increase of pitting corrosion potential and passive region. It could be concluded that the duplex surface treatment improved the passive film stability and corrosion resistance. The corrosion behavior of metallic materials can be strongly affected by the chemical composition and electronic states of the surface layer^20^. The mechanism of passivation on 316L will be different at high temperature comparing with that at room temperature, since the oxidation rate increases dramatically with increasing temperature. In order to characterize the microstructure of the passive films formed on the surface of reference samples and sample with duplex process, experiments of XPS and SIMS were carried out and the results were presented in Figs 6 and 7 respectively.

According to the binding energy (BE), Cr 2p3/2 peaks could be decomposed into three components: one located at a BE of 574.4 ± 0.2 eV, and two other ones located at BEs of 576.6 ± 0.2 eV and 577.4 ± 0.3 eV. Comparing with the NIST database^28^, La Surface database^29^ and other researches^30–32^, the signal at the highest BE was assigned to Cr^3+^ in Cr(OH)_3_, the signal at 576.6 ± 0.2 eV to Cr^3+^ in Cr_2_O_3_ or spinel oxides containing Cr (NiCr_2_O_4_, NiCrFeO_4_, and so on), the signal at 574 ± 0.2 eV to Cr^0^. As shown in Fig. 6, the peak intensity of Cr(OH)_3_ reduced gradually and the fraction of Cr_2_O_3_ increased with SMAT treatment. For example, The content of Cr_2_O_3_ in the passive film increased from 42.4% ± 2.1% (untreated sample) to 64.9% ± 5.4% (treated samples), shown in Table 2 and Fig. 6. The distribution of oxide and hydroxide in the passive film, formed on 316L without and with the duplex treatment, was identified by SIMS. The spectra in Fig. 7 showed that the oxide film consisted of Cr^3+^, Fe^2+^, Fe^3+^, Ni^2+^, OH^−^ and O^2−^ ions. And it can be observed that the Cr/(Cr^+^, Ni^+^ Fe^+^) value is much higher for treated sample comparing with the reference sample. Depth profile analyses suggested that the oxide film likely consisted of Cr-rich layer with the duplex treatment, as during SMAT process, high-density dislocations and grain boundaries in treated layer enhanced the outward diffusion of Cr to form a protective Cr-rich layer near the surface.

**Table 2:** The Peak Fitting Parameters for XPS.

		FWHM	Peak Area(a.u)	Gaussian-Lorentizian%	Percentage in total
Reference sample	Cr_2_O_3_	1.66	1320 ± 66	20%	40.6% ± 2.0%
	Cr(OH)_3_	1.8	1790 ± 90	20%	55% ± 2.8%
	Cr^0^	2.4	140 ± 9	20%	4.4 ± 0.3%
SMAT + Nitriding	Cr_2_O_3_	1.6	6650 ± 550	20%	61.6% ± 5%
	Cr(OH)_3_	1.92	3600 ± 314	20%	33.3% ± 2.9%
	Cr^0^	2.6	540 ± 58	20%	5.1 ± 0.5%

Both XPS and SIMS results indicate that the duplex process leads to a significant increase in the amount of Cr oxide in the passive film. This Cr_2_O_3_ has been reported to be the stable crystalline form of chromium oxide^33^, which can passivate the surface in the steady state at high temperature. This result agreed with other reports^34,35^. As the Cr-rich layer was stable, the corrosion resistance has been improved, as shown in the electrochemical measurement.

This work investigated the effect of the duplex SMAT and nitriding treatment on mechanical properties and corrosion performance of stainless steel 316L both at room temperature and at 300 °C in the simulated steam generator (SG) condition. The experimental results showed that the duplex process fabricates a nanostructured layer on the surface and resulted in an increase of the microhardness to 1050 HV. The corrosion resistance after the duplex SMAT and nitriding treatment appeared to be improved at room temperature. And in the simulated SG condition at 300 °C, a higher pitting potential and a wider passive region has been observed because of the increase of Cr_2_O_3_ in the passive layer on the surface of treated 316L. The better corrosion resistance at both conditions contributes not only to the releasing of surface energy during the nitriding process but also to large amount of defects induced by SMAT, which providing high density of nucleation sites and fast diffusion paths for N and Cr thus leading to the rapid formation of a protective layer.

## Methods

The alloys 316L samples (50 × 25 × 2 mm^3^ in size) were used for the experiments and chemical composition in weight percent is 0.08C, 0.045P, 0.03S, 16Cr, 12Ni, 2Mn, 1Si, 2.0Mo and the balance is Fe. Before SMAT, the specimen’s surfaces were polished with silicon carbide papers and then annealed in vacuum at 1273 K for 120 min for diminishing the effect of mechanical processing and obtaining homoge neous coarse grains. After degreased using acetone, the 316L samples were subjected to SMAT on both surfaces with stainless steel balls of ф3 mm in diameter at room temperature (25 °C) for 60 mins at a fixed frequency of 20 kHz. The details of the SMAT process were described in an earlier paper^20^. Then the treated samples were nitrided in a flowing NH_3_ (99.9995%) at a pressure of 1 atm at 400 °C for 6 h. TEM was performed to examine the microstructure of the nanocrystalline layer utilizing JEOL JEM 2010 with 200 kV accelerating voltage. The phase content and residual stress of reference, nitrided samples without and with SMAT process were investigated by XRD with a Philips X’pert diffractometer using Cu K_._ in the range of 20–80°, the measurements being performed with a continuous scanning mode at a rate of 0.02°/s. While, the residual stress with removal of the surface layer by layer using electro-polishing was determined by the sin2ψ method. Peaks measured at higher 2θ were chosen to acquire accurate information on residual stress. The hardness distribution along the cross-section of the reference, nitrided samples without and with SMAT were measured using the Future Tech Vicker’s hardness tester under an applied load of 25 g. Potentiodynamic polarization measurements at room temperature were carried out using CHI660 electrochemical workstation at room temperature, and a 3.5 wt% NaCl solution was used as the corrosive medium. A saturated calomel electrode (SCE) was used as a reference electrode, and a platinum plate was used as a counter electrode. The potential range was from −1000 mV vs. SCE to 1000 mV vs. SCE at a scan rate of 100 mV/min. While the electrochemical behavior of samples without and with duplex treatment at high temperature was studied in simulated SG crevice chemistries at 300 °C, an Ag/AgCl electrode was used as the reference electrode, and this reference electrode was immersed in 0.65 M KCl solution, which was the same as the Cl^−^ concentration in the testing solutions. The potential report has been transformed into the SCE scale, the potential range was from rom −1000 mV vs. SCE to 200 mV vs. SCE. The chemical composition of the oxide films of the samples after corrosion was investigated using XPS, an Axis-ULTRA (Kratos Analytical) spectrometer controlled by a SUN workstation which was equipped with a monochromatic X-ray source (Al Kα, hυ = 1486.6 eV), at steps of 0.1 eV using 20 eV pass energy; the SIMS analysis was performed to investigate the concentration depth profile along the cross-section of the passive film on the surface of the samples without or with treatment, the sputtering source was Cs^+^, operated at 2 kV.
